# Comparative effectiveness of abatacept versus tocilizumab in rheumatoid arthritis patients with prior TNFi exposure in the US Corrona registry

**DOI:** 10.1186/s13075-016-1179-7

**Published:** 2016-12-01

**Authors:** Leslie R. Harrold, George W. Reed, Daniel H. Solomon, Jeffrey R. Curtis, Mei Liu, Jeffrey D. Greenberg, Joel M. Kremer

**Affiliations:** 1 Department of Medicine, University of Massachusetts Medical School, 55 Lake Avenue North, AC7-201, Worcester, MA 01655 USA; 2 Corrona, LLC, Southborough, MA USA; 3 University of Alabama at Birmingham, Birmingham, AL USA; 4 NYU School of Medicine, New York, NY USA; 5 Albany Medical College, Albany, NY USA; 6 The Center for Rheumatology, Albany, NY USA; 7 Brigham and Women’s Hospital, Boston, MA USA

**Keywords:** Rheumatoid arthritis, Disease-modifying anti-rheumatic drugs (biologic), Tocilizumab, Abatacept, Treatment

## Abstract

**Background:**

We compared the effectiveness of abatacept (ABA) vs tocilizumab (TCA) in tumor necrosis factor inhibitor (TNFi) experienced patients.

**Methods:**

We identified rheumatoid arthritis (RA) patients from a large observational US cohort (1 January 2010–31 May 2014) who had discontinued at least one TNFi and initiated ABA or TCZ in moderate or high disease activity based on the Clinical Disease Activity Index (CDAI) and had no prior exposure to the comparator drug. Using propensity score matching (1:1) stratified by prior TNF use (1 TNFi vs ≥2 TNFis), effectiveness at 6 months after initiation was evaluated. Mean change in CDAI over 6 months following initiation was the primary outcome, with secondary outcomes of achievement of low disease activity/remission (CDAI ≤ 10) and mean change in modified Health Assessment Questionnaire (mHAQ) score.

**Results:**

The 264 pairs of propensity score-matched ABA and TCZ initiators were well matched with no substantial differences in the baseline characteristics, defined as standardized differences >0.1 in the stratification. Both treatment groups had similar mean change in CDAI at 6 months (–11.3 in ABA vs –9.9 in TCZ; mean difference –1.27, 95% CI –3.65, 1.11). Similar proportions of both treatment groups achieved low disease activity/remission (adjusted odds ratio for ABA vs TCZ 0.99, 95% CI 0.69, 1.43). Mean change in mHAQ was –0.12 in ABA initiators vs –0.11 in TCZ initiations (mean difference –0.01, 95% CI –0.09, 0.06).

**Conclusions:**

Patients receiving either ABA or TCZ had substantial improvement in clinical disease activity. In this propensity score-matched sample, similar outcomes were observed for both treatment cohorts.

**Electronic supplementary material:**

The online version of this article (doi:10.1186/s13075-016-1179-7) contains supplementary material, which is available to authorized users.

## Background

Rheumatoid arthritis (RA) is a chronic, inflammatory disease characterized by persistent synovitis and associated with pain, functional disability, and decreased quality of life as well as increased risk of death affecting an estimated 1.3 million Americans [1, 2]. The goal of therapy is to reduce disease activity and improve clinical outcomes. The current treatment paradigm is to first use conventional disease-modifying anti-rheumatic drugs (cDMARDs) followed by step-up to combination cDMARD therapy or initiation of a biologic [3–5]. Typically, a tumor necrosis factor inhibitor (TNFi) is the first biologic class initiated [6]. While this class of drugs is associated with improvement in the signs and symptoms of RA, it has been shown both in large randomized controlled trials (RCTs) and in everyday clinical practice that as many as 30–40% of patients develop an inadequate response to TNFis [7–10]. This inadequate response may be related to either primary nonresponse (lack of response after initiation) or a secondary nonresponse which is treatment failure due to drug resistance or intolerance.

However, there is conflicting information regarding which should be the next agent to manage a patient who has had an inadequate response to a TNFi. There have been inconsistent results regarding the benefits of changing mechanism of action in observational data as a general approach, or whether targeting a specific pathway after failure of a TNFi will optimize outcomes. For example, improved outcomes were demonstrated in comparisons of rituximab vs a subsequent TNFi [11–14] but not in abatacept (ABA) initiators vs a subsequent TNFi [15]. More recently a RCT found greater effectiveness with use of non-TNFi biologics as compared with a second anti-TNF drug in TNF inadequate responders [16].

Given the absence of head-to-head RCTs comparing the non-TNFi biologics in patients with inadequate response to an anti-TNF agent, comparative effectiveness studies using observational data from registries can be employed [17]. To address the limitations of observational studies, such as selection bias, propensity score methodology is commonly employed [15, 18, 19]. We used propensity score matching to compare the clinical effectiveness of tocilizumab (TCZ) vs ABA among RA patients with previous anti-TNF exposure in a large US cohort of RA patients using the Consortium of Rheumatology Researchers of North America (Corrona) registry. Specifically, we sought to compare change in disease activity, achievement of low disease activity (LDA), and change in function over 6 months.

## Methods

### Data source

The Corrona registry is an independent, prospective, observational cohort of patients with RA recruited at >160 private and academic practice sites across 40 states in the United States; additional details have been published previously [20]. As of 30 May 2015, data on more than 40,989 patients with RA have been collected. Corrona’s database includes information about 303,260 patient visits and approximately 130,699 patient-years (PY) of follow-up observation time, with a mean time of patient follow-up of 3.87 years (median, 2.99 years). Data are collected from both patients and their treating rheumatologists, who gather information on disease duration, prognosis, disease severity and activity, medical comorbidities, use of medications including biologics, cDMARDs, and prednisone, and adverse events. Follow-up assessments are requested at least as often as every 6 months and completed during routine clinical encounters. For this national study, approvals for data collection and analyses were obtained from a central institutional review board (New England Institutional Review Board) for private practice sites participating within Corrona. For the <20% of sites that are affiliated with an academic medical center, local institutional review boards were the Institutional Review Board of record.

### Study population

**Fig. 1:**
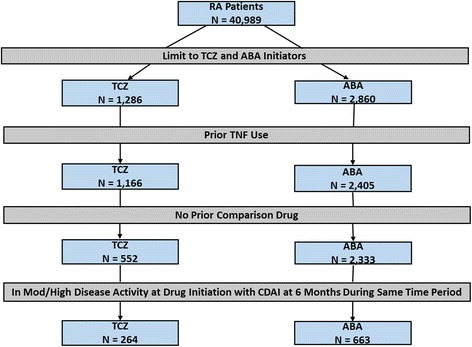
Flow diagram for tocilizumab (*TCZ*) and abatacept (*ABA*) initiators based on the study criteria. No prior comparison drug means the TCZ patients had no prior exposure to ABA and the ABA patients had no prior exposure to TCA. *CDAI* Clinical Disease Activity Index, *Mod* moderate, *RA* rheumatoid arthritis, *TNF* tumor necrosis factor

Data were collected from patients with RA from the Corrona registry who initiated TCZ or ABA on or after 1 January 2010. The study population was limited to patients who had received ≥1 TNFi and no prior use of the comparator drug. Patients must have had the following data available to be included in the study: date of the ABA or TCZ initiation; follow-up visit at 6 months (window 5–10 months); and Clinical Disease Activity Index (CDAI) measurements at baseline and 6-month follow-up visit. For patients whose initiation occurred between visits, a prior visit (within 4 months of initiation) was used for baseline characteristics. Patients in LDA or remission at initiation based on the CDAI were excluded from the study (Fig. 1). All patients provided written informed consent prior to participation.

### Measures and data collection

Data from Corrona were collected during the study period (1 January 2010–31 May 2014) from physician and patient questionnaires completed during routine clinical encounters. Biologic, cDMARD, and prednisone use was recorded at the time of the clinical encounter as well as 28-joint tender and swollen joint counts, physician and patient global assessments of disease activity, patient assessment of pain, and modified Health Assessment Questionnaire (mHAQ) score assessing physical function [21]. Data on demographics, insurance status, comorbid conditions, RA disease characteristics, and RA medications were available for ≥99% of patients.

### Drug exposure cohorts

To balance for predisposing factors that may increase a patient’s likelihood of receiving either ABA or TCZ, a propensity score [22, 23]—or the probability of treatment selection—was calculated for each eligible patient using baseline (at the time of drug initiation) patient demographics (age, sex, and insurance type), disease characteristics (rheumatoid factor seropositivity, American College of Rheumatology functional class, patient and provider global assessments, disease activity, and functional status based on the mHAQ), comorbidities (history of serious infectious event), and concurrent medications (prednisone). The ABA and TCZ patients were stratified by 1 TNFi vs ≥2 prior TNFis and then matched within each stratum based on the propensity score estimated within each strata without replacement using calipers of 0.01. The resulting stratified-matched population resulted in 264 matched pairs.

### Study outcomes

Responsiveness to medication treatment was defined based on mean change in CDAI as our primary outcome (e.g., follow-up CDAI – baseline CDAI). As secondary outcomes, we examined the proportion of patients who achieved LDA/remission (CDAI score ≤ 10) and change in the mHAQ score at 6 months. Acceleration and deceleration of prednisone dosing was examined (both initiation/discontinuation as well as dose increase/decrease) over the time period. We utilized the same parameters for cDMARD initiation and discontinuation over the study period.

### Analysis and statistical methods

Patients were included regardless of switching or discontinuation of the medication without initiating another biologic among the two comparator groups. Baseline patient demographics and clinical and disease characteristics were compared between the two drug-exposure cohorts, and standardized differences were estimated. Standardized differences provide a measure of clinically important differences (even if there are no statistically significant differences). A standard difference < 0.1 has been taken to indicate a negligible difference in the mean or prevalence of a covariate between treatment groups [22]. Response was defined based on primary and secondary outcomes at 6 months regardless of continuation of initial treatment. For those patients who discontinued the drug without initiating another biologic, the observations at the 6-month visit were used. For those who switched to another agent, the last observation prior to the switch was used as the primary analytic approach. Additionally, we imputed nonresponse for patients who switched biologics. However, because the results were similar to the last observation carried forward approach, we do not present the results for the imputed nonresponse in this article. Descriptive statistics were used to examine rates of response at 6 months.

Linear and logistic regression models were fit to estimate the adjusted mean difference and odds ratios (ORs) as appropriate with 95% CIs comparing response rates in TCZ initiators with ABA initiators. The resulting regression models were adjusted considering the matched-pair as a random effect (e.g., patient clustered within the matched pair). Using the stratified-matching strategy, all of the baseline characteristics had standardized differences < 0.1. No additional covariates were therefore used in the models. Safety outcomes were also compared between the two groups. Specifically, we assessed all cancers (excluding nonmelanoma skin cancer), infections (all infections and serious infections, defined as those infections requiring hospitalization or intravenous antibiotics), and cardiovascular events (including myocardial infarction, stroke, acute coronary syndrome, coronary artery disease, congestive heart failure, peripheral arterial disease, and hypertension) reported by the providers over the 6-month period. We used a time to event approach to obtain the rate of events for each category of adverse event based on the available person-time.

## Results

### Baseline demographics

A total of 663 ABA and 264 TCZ initiators met the inclusion criteria prior to implementation of the propensity scores (Fig. 1). Over the 6-month period most initiators stayed on the medication (92.3% ABA and 89.0% TCZ were persistent), with switching (5.7% ABA and 7.6% TCZ) and discontinuation without initiating another biologic (2.0% ABA and 3.4% TCZ) occurring in the minority of patients. Comparison of the two treatment arms revealed differences (standardized differences > 0.1) in terms of the proportion of women, history of prior infections, patient and global assessments of disease activity, and number of prior biologics (Table 1). The two treatment arms were then stratified and propensity score matched (1:1), resulting in 264 participants each in the two arms (44.7% with one prior TNFi and 55.3% with ≥2 prior TNFis). There were no substantial differences in the baseline characteristics of the two groups defined as standardized differences > 0.1 in the stratified matched sample, as shown in Table 1, and thus adjusted analyses were not required. Overall persistency was similar with the median time from initiation to discontinuation 17.7 months (13.6, 23.9) in ABA users as compared with 17.9 months (13.7, 27.2) in TCZ users (*p* = 0.81). Most patients were female, in their mid-50s, with approximately a decade of disease duration, and had high disease activity at the time of drug initiation with 10 tender joints and seven swollen joints. Just over half (52%) of both groups were receiving methotrexate (with a mean dose of 17.1 mg (SD 5.1) in ABA users and 18.7 mg (SD 5.1) in TCZ users). Similar percentages of patients remained on drug (95.5% ABA vs 89.0% TCZ), switched biologics (6.8% ABA vs 7.6% TCZ), or discontinued biologics altogether (2.7% ABA vs 3.4% TCZ) over the study period. At initiation, 85% of ABA users received intravenous infusions and 15% received subcutaneous medication. In the TCZ initiators, 83% received intravenous infusions and 17% received subcutaneous injections.

**Table 1:** Patient demographics and clinical characteristics at baseline by tocilizumab and abatacept initiators

Characteristic	Unmatched			Matched		
	TCZ Initiators	ABA Initiators	Standardized difference^a^	TCZ Initiators	ABA Initiators	Standardized difference^a^
	(*n* = 264)	(*n* = 663)	ABA – TCZ	(*n* = 264)	(*n* = 264)	ABA – TCZ
Female, *n* (%)	197 (74.6)	547 (82.9)	0.2	197 (74.6)	197 (74.6)	0
Age, mean ± SD	56.6 ± 12.4	57.3 ± 12.3	0.05	56.6 ± 12.4	56.6 ± 12.2	–0.01
Race: White, *n* (%)	201 (76.1)	513 (77.4)	0.03	201 (76.1)	211 (79.9)	0.09
Smoker, *n* (%)						
Never	119 (45.1)	305 (46.1)	0.07	119 (45.1)	115 (43.6)	0.03
Previous	90 (34.1)	237 (35.8)		90 (34.1)	94 (35.6)	
Current	55 (20.8)	119 (18.0)		55 (20.8)	55 (20.8)	
Insurance^b^, *n* (%)						
Private	205 (77.6)	516 (77.8)	0.01	205 (77.6)	193 (73.1)	–0.1
Medicaid	12 (4.5)	34 (5.1)	0.03	12 (4.5)	14 (5.3)	0.03
Medicare	96 (36.4)	210 (31.7)	–0.1	96 (36.4)	92 (34.8)	–0.03
None	3 (1.1)	10 (1.5)	0.03	3 (1.1)	6 (2.2)	0.09
Duration of RA, mean ± SD	10.7 ± 8.8	11.1 ± 9.3	0.03	10.7 ± 8.8	10.3 ± 10.0	–0.03
RF/CCP seropositivity, *n* (%)	127 (76.5)	291 (74.1)	–0.06	127 (76.5)	135 (73.7)	–0.06
History of comorbidities, *n* (%)						
Cardiovascular	43 (16.3)	88 (13.3)	–0.08	43 (16.3)	41 (15.5)	–0.02
Malignancy	14 (5.3)	37 (5.6)	0.01	14 (5.3)	16 (6.1)	0.03
Serious infection	18 (6.8)	67 (10.1)	0.12	18 (6.8)	23 (8.7)	0.07
Diabetes	26 (9.9)	64 (9.7)	–0.01	26 (9.9)	33 (12.5)	0.08
mHAQ, mean ± SD	0.7 ± 0.5	0.6 ± 0.5	–0.16	0.7 ± 0.5	0.7 ± 0.5	0.02
CDAI, mean ± SD	27.8 ± 12.1	27.1 ± 11.9	–0.05	27.8 ± 12.1	28.1 ± 12.8	0.04
Tender joints, mean ± SD	10.3 ± 7.6	10.0 ± 7.2	–0.05	10.3 ± 7.6	10.4 ± 7.5	0.001
Swollen joints, mean ± SD	7.0 ± 4.9	7.4 ± 5.6	0.08	7.0 ± 4.9	7.3 ± 5.8	0.05
Physician global, mean ± SD	47.2 ± 20.4	43.8 ± 20.5	–0.16	47.2 ± 20.4	47.7 ± 20.9	0.02
Patient global, mean ± SD	57.0 ± 23.6	53.2 ± 23.9	–0.16	57.0 ± 23.6	56.8 ± 23.3	0
Patient pain, mean ± SD	58.6 ± 24.6	55.9 ± 25.4	–0.1	58.6 ± 24.6	58.3 ± 25.5	–0.01
ACR functional status, *n* (%)						
I	55 (20.8)	200 (30.2)		55 (20.8)	48 (18.2)	
II	130 (49.2)	306 (46.2)	0.23	130 (49.2)	138 (52.3)	0.07
III/IV	79 (29.0)	157 (23.7)		79 (29.9)	78 (29.5)	
Prednisone use, *n* (%)						
None	166 (63.4)	415 (63.1)		166 (63.4)	168 (63.9)	
<10 mg	53 (20.2)	144 (21.9)	0.04	53 (20.2)	50 (18.8)	0.03
≥10 mg	43 (16.4)	99 (15.1)		43 (16.4)	44 (16.5)	
Number of prior biologics used, *n* (%)						
1	99 (37.5)	261 (39.4)	0.12	99 (37.5)	109 (41.3)	–0.08
2	94 (35.6)	257 (38.8)		165 (62.5)	155 (58.7)	
3+	71 (26.9)	145 (21.8)				
Concomitant use of cDMARD, *n* (%)	180 (68.2)	456 (68.8)	0.01	180 (68.2)	179 (67.8)	–0.01

^a^A standard difference < 0.1 has been taken to indicate a negligible difference in the mean or prevalence of a covariate between treatment groups

^b^patients may have more than 1 type of insurance

*ABA* abatacept, *ACR* American College of Rheumatology, *CCP* cyclic citrullinated peptide, *CDAI* Clinical Disease Activity Index, *cDMARD* conventional disease-modifying anti-rheumatic agent, *mHAQ* modified health assessment questionnaire, *RA* rheumatoid arthritis, *RF* rheumatoid factor, *TCZ* tocilizumab

### Outcomes at 6 months

Mean change in CDAI at 6 months in the stratified propensity score-matched treatment groups were similar: –11.3 ± 14.7 in the ABA initiators vs –9.9 ± 14.1 in the TCZ initiators (Table 2). The difference was –1.27 (95% CI –3.65 to 1.11) for ABA use as compared with TCZ. Achievement of LDA/remission occurred in 1/3 of patients in both groups with an OR of 0.99 (95% CI 0.69, 1.43). Similarly, the change in mHAQ was –0.12 ± 0.42 for ABA and –0.11 ± 0.49 for TCZ, resulting in a difference of –0.01 (95% CI –0.09, 0.06).

**Table 2:** Comparison between tocilizumab and abatacept initiators in terms of mean change in CDAI, mean change in mHAQ, and achievement of low disease activity from baseline to 6 months

Outcome	Tocilizumab initiators (*n* = 264)	Abatacept initiators (*n* = 264)	Unadjusted difference^a^ abatacept vs tocilizumab	
Continuous	Mean ± SD	Mean ± SD	β (95% CI)	*p* value
Change in CDAI	*n* = 259, –9.9 ± 14.1	*n* = 257, –11.3 ± 14.7	–1.27 (–3.65, 1.11)	0.30
Change in mHAQ	*n* = 263, –0.11 ± 0.49	*n* = 264, –0.12 ± 0.42	–0.01 (–0.09, 0.06)	0.83
Binary	Response rate	Response rate	OR (95% CI)	*p* value
Achievement of LDA	*n* = 259	*n* = 257		
(CDAI at time of switch was imputed for switchers)	86 (33.2%)	85 (33.1%)	0.99 (0.69, 1.43)	0.97

^a^Matched pair as the random effect

*CDAI* Clinical Disease Activity Index, *CI* confidence interval, *LDA* low disease activity, *mHAQ* modified Health Assessment Questionnaire, *OR* odds ratio

### Use of concomitant prednisone and cDMARDs

There were no differences between the groups in terms of change in prednisone dose. Most patients in both treatment groups had no change in prednisone use over the study period (68.7% ABA vs 67.2% TCZ). Dose increases (either initiation or an increase of prednisone dose) occurred in 12.6% of ABA vs 13.7% of TCZ users while dose reduction (including discontinuation) occurred in 18.7% ABA and 19.1% of TCZ users. At 6 months, 44.4% of ABA and 54.2% of TCZ users on prednisone were receiving ≤5 mg/day (*p* = 0.21). Similarly, cDMARD initiations (14.4% ABA vs 11.4% TCZ) and discontinuations (15.2% ABA vs 13.3% TCZ) were not different in the two groups.

### Safety

The rates of AEs in the two populations are reported in Table 3. The standardized rates per 100 person-years for cancer, serious infection, and cardiovascular events in ABA vs TCZ users were 1.2 vs 0.6, 2.5 vs 1.9, and 1.9 vs 2.5, respectively. No significant differences were observed between the two groups.

**Table 3:** Rates of cancers, infection (overall and serious infections), and cardiovascular adverse events

	Adverse event rates, events/100 PY			
	Tocilizumab	Abatacept	Ratio (95% CI) of rates tocilizumab/abatacept	*p* value
Cancer^a^	0.61 events/155.2 PY	1.22 events/157.2 PY	0.5 (0.1, 9.7)	0.63
Infection				
All events	54.373 events/135.4 PY	57.179 events/138.3 PY	0.9 (0.7, 1.3)	0.72
Serious infections	1.93 events/155.2 PY	2.54 events/157.7 PY	0.7 (0.1, 4.5)	0.74
Cardiovascular	2.54 events/154.6 PY	1.93 events/156.7 PY	1.3 (0.2, 9.2)	0.71

^a^Excluding nonmelanoma skin cancer

*CI* confidence interval, *PY* person-years

## Discussion

Using data from Corrona, a large US-based RA registry, we compared the clinical effectiveness of TCZ vs ABA among TNFi-experienced patients with moderate-to-high disease utilizing patients from real-world rheumatology practices. We compared change in disease activity, achievement of LDA/remission as well as meaningful improvement in function in the two treatment groups. Both treatments were effective for the primary outcome because the minimally clinical important difference for change in CDAI is a reduction of 6 points for moderate disease activity and 12 points for high disease activity [24]. Furthermore, we conducted a post-hoc analysis to evaluate whether the dose of TCZ contributed to the results. Specifically, there was no difference in terms of change in CDAI, change in mHAQ, or achievement of LDA/remission among those on 4 mg/kg at the end of the study period as compared with those who had escalated to 8 mg/kg (*n* = 182, 69%). Additionally, the safety of the two agents was comparable over the study time period.

Our results are similar to an observational cohort from Japan [19], in which investigators examined outcomes in 102 matched pairs of patient treated with TCZ at 8 mg/kg every 4 weeks vs ABA dosed based on weight (500 mg for patients < 60 kg, 750 mg for patients 60–100 kg, and 1000 mg for patients > 100 kg) at weeks 0, 2, and 4 and then every 4 weeks after. They found no significant difference in retention rates between the two agents, which is consistent with our findings (Additional file 1: Table S1). When evaluating efficacy over 52 weeks using the SDAI they identified no differences between the two agents, with approximately 46–50% achieving LDA/remission at 6 months and 47–55% at 12 months. These rates are slightly higher than our study, but this may be in part be due to differences in the outcome measure used, patient characteristics, and study design. Of note, the patients in our cohort had higher baseline disease activity based on the CDAI. Furthermore, Corrona’s registry reflects real-world practice. Physicians may thus administer medications at doses and frequencies different from the package labeling (e.g., holding doses for procedures or hospitalizations). Additionally, we included patients in the analyses regardless of whether they switched to another biologic or discontinued the agent without starting a new biologic. With respect to functional impairment, both studies showed no difference between the treatment groups (ours using mHAQ scores and the Japanese cohort using HAQ-DI scores).

We additionally examine the impact of concomitant RA medications on our findings. This included an evaluation as to whether there were differences in the use of prednisone (both initiation and dose escalation) and cDMARDs (initiation and discontinuation) between the two groups. No such differences were found (Additional file 1: Tables S2 and S3). We also explored whether our selection approach influenced the results. We found similar outcomes in ABA patients who were not selected for inclusion into the study (because of receipt of the medication prior to the availability of TCA) to the ABA patients who were included.

We believe that comparative effectiveness studies of non-TNF biologics are needed for practicing rheumatologists when trying to decide which agents to prescribe in their patients with prior TNFi exposure. Rheumatologists want to prescribe the right drug to the right patient at the right time. Providing comparative data with accompanying safety information is essential for rheumatologists in order to facilitate patients achieving the best clinical outcomes. Given the lack of head-to-head RCTs, observational studies are the best approach for evaluating comparative treatment outcomes. Using “best practice” approaches, propensity score methodology was used specifically due to selection bias. Based on these findings, both agents were associated with substantial improvement in RA symptoms. The individual choice of which agent to prescribe will likely be informed based on patient characteristics, pharmacy benefit plan, and patient preference.

This study had several strengths. It is the largest known comparative effectiveness study of ABA vs TCZ in the United States and included a nationwide sampling of patients with RA. The all-comers study design recruited individuals from multiple rheumatology centers, resulting in a range of patients with real-world disease activity and comorbidities not often seen in RCTs. We also evaluated effectiveness by examining response in all patients regardless of continuation of initial treatment. This has been shown to provide a conservative estimate [15]. Additionally, we examined comparative effectiveness broadly with evaluation of response using the CDAI, function using the mHAQ, and safety between the two drug-exposure cohorts because all three elements are needed for clinical decision-making.

This study also had some limitations related to the challenges of operationalizing available, real-world data, and applying analytical methods conservatively. As with any registry, there is a concern that patient enrollment may not reflect the type of patients observed elsewhere in general practice. However, a previous study [25] demonstrated that Corrona RA patients enrolled in Medicare shared similar demographic and clinical characteristics to the national cohort of RA patients based on Medicare rheumatology-based claims. Of note, we only had sufficient sample size to explore 6-month outcomes. Potentially a longer time frame may have demonstrated differences. Also, as in any observational study, bias is a concern because physicians prescribe therapies based on the patient’s profile and treatment selection is not random. To overcome this limitation, we matched by propensity score and stratified by prior TNFi. However, this method does not address unmeasured confounders. We did include patients in the analyses who discontinued ABA or TCZ (<4% in each group) without initiating a subsequent biologic because the reason for discontinuation was not clear (e.g., drug holiday due to a good response to the medication, side effects, out-of-pocket costs, etc.). Because there was no significant difference between groups in discontinuation rates, however, the outcome of the study (i.e., no difference in clinical response between the two drugs) would not be altered if we excluded these patients from the analyses. Because of sample size considerations, sensitivity analyses were not performed based on reasons for discontinuation of the prior TNFi, although other studies have shown that this can influence treatment response [26].

## Conclusions

Among patients with prior exposure to TNFi, use of ABA and TCZ both resulted in improved disease activity based on the MCID of the CDAI and function as measured with the mHAQ. Furthermore, the safety profiles of the two treatments were similar over the 6-month time frame. Based on these findings, the results suggest that both agents would be appropriate choices as the next therapeutic agent in patients with moderately-to-severely active RA with prior exposure to TNFis. Further analyses are necessary to better identify the patient subsets likely to respond to a particular agent.

## Additional file

Additional file 1: Table S1 describes the rates of remaining on drug, switching, and discontinuing without starting a new biologic in the TCZ and ABA groups before and after match. Table S2 presents the distribution of prednisone dose increases and decreases between the matched TCA and ABA initiators based on the baseline prednisone usage. Table S3 presents rates of discontinuation and initiation of cDMARDs over the 6-month follow-up. (DOCX 14 kb)

## Abbreviations

AE: Adverse event
CDAI: Clinical Disease Activity Index
cDMARD: Conventional disease-modifying anti-rheumatic drug
CI: Confidence interval
LDA: Low disease activity
mHAQ: Modified Health Assessment Questionnaire
OR: Odds ratio
RA: Rheumatoid arthritis
RCT: Randomized controlled trial
RF: Rheumatoid factor
TNFi: Tumor necrosis factor inhibitor
