# Supplementary material for: Comparative effectiveness of abatacept versus tocilizumab in rheumatoid arthritis patients with prior TNFi exposure in the US Corrona registry
Source: Arthritis Res Ther. 2016 Dec 1;18:280. doi: 10.1186/s13075-016-1179-7 (PMC5134270; doi:10.1186/s13075-016-1179-7)
Supplement: Additional file 1: — Table S1 describes the rates of remaining on drug, switching, and discontinuing without starting a new biologic in the TCZ and ABA groups before and after match. Table S2 presents the distribution of prednisone dose increases and decreases between the matched TCA and ABA initiators based on the baseline prednisone usage. Table S3 presents rates of discontinuation and initiation of cDMARDs over the 6-month follow-up. (DOCX 14 kb) [file 13075_2016_1179_MOESM1_ESM.docx]

**Supplementary material:**

**Table S1. The rates of remaining on drug, switching and discontinuing without**

**starting a new biologic in Tocilizumab (TCZ) and Abatacept (ABA) groups**

**before match and after match**

|  |  | TCZ Initiators  N = 264 | ABA Initiators  N =663 |
| --- | --- | --- | --- |
| Before PS Matching | Remain on Drug | 235(89.0%) | 612(92.3%) |
|  | Switch to another biologic | 20 (7.6%) | 38(5.7%) |
|  | Discontinue drug but no switch | 9 (3.4%) | 13(2.0%) |
|  |  |  |  |
|  |  | TCZ Initiators  N = 264 | ABA Initiators  N =264 |
| After PS Matching | Remain on Drug | 235(89.0%) | 239 (90.5%) |
|  | Switch to another biologic | 20 (7.6%) | 18 (6.8%) |
|  | Discontinue drug but no switch | 9 (3.4%) | 7 (2.7%) |

**Table S2. The distribution of dose increase and decrease between the matched tocilizumab (TCA) and abatacept (ABA) initiators with baseline prednisone dose information. No significant difference was found between the groups. (P= 0.91)**

|  | TCZ Initiators  N = 262 | ABA Initiators  N =262 |
| --- | --- | --- |
| Dose Increase | 36(13.7%) | 33(12.6%) |
| Initiated prednisone | 21 (8.0%) | 22(8.4%) |
| Increase dose | 15 (5.7%) | 11(4.2%) |
| Dose reduction | 50(19.1%) | 49(18.7%) |
| Discontinue prednisone | 34(12.9%) | 33(12.6%) |
| Decrease dose | 16(6.1%) | 16(6.1%) |
| No change | 176(67.2%) | 180(68.7%) |

**Table S3. Discontinuation and initiation of conventional disease modifying anti-rheumatic drugs (cDMARDs) over the 6 months follow-up.**

|  | TCZ Initiators  N = 264 | ABA Initiators  N =264 |
| --- | --- | --- |
| cDMARD discontinuation | 35(13.3%) | 40(15.2%) |
| cDMARD initiation | 30 (11.4%) | 38 (14.4%) |
